# The Hydroxytyrosol Induces the Death for Apoptosis of Human Melanoma Cells

**DOI:** 10.3390/ijms21218074

**Published:** 2020-10-29

**Authors:** Francesca Costantini, Caterina Di Sano, Giovanna Barbieri

**Affiliations:** Istituto per la Ricerca e l’Innovazione Biomedica (IRIB), Consiglio Nazionale delle Ricerche (CNR), Via Ugo La Malfa 153, 90146 Palermo, Italy; francesca.costantini@irib.cnr.it (F.C.); caterina.disano@irib.cnr.it (C.D.S.)

**Keywords:** melanoma, hydroxytyrosol, cellular growth, apoptosis, ROS

## Abstract

Melanoma is the most aggressive form of skin cancer and one of the most treatment-refractory malignancies. In metastatic melanoma cell lines, we analysed the anti-proliferative and pro-apoptotic potentials of a phenolic component of olive oil, the hydroxytyrosol. In particular, through MTS assay, DeadEnd™ Colorimetric TUNEL assay, Annexin V binding and PI uptake, western blot experiment, intracellular reactive oxygen species (ROS) analysis, and the cell colony assay, we showed that the hydroxytyrosol treatment remarkably reduces the cell viability inducing the death for apoptosis of melanoma cells. Moreover, we showed that the hydroxytyrosol treatment of melanoma cells leads to a significant increase of p53 and γH2AX expression, a significant decrease of AKT expression and the inhibition of cell colony formation ability. Finally, we propose that the increased amount of intracellular reactive oxygen species (ROS) that may be related to the regulation of the pathways involved in the activation of apoptosis and in the inhibition of melanoma growth could be the strategy used by hydroxytyrosol to exert its functions in melanoma. Therefore, for its role in melanoma growth inhibition, the hydroxytyrosol treatment could deeply interfere with melanoma progression as a promising therapeutic option for the treatment of this highly invasive tumour.

## 1. Introduction

Melanoma is one of the most aggressive cancers worldwide and the deadliest form of skin cancer whose incidence rate, unfortunately, is increasing rapidly in western populations [1]. The transformation of melanocytes into melanoma cells is a multistep process involving mutations of survival molecules, inhibition of apoptotic pathways, and increase of cellular migration [2]. Indeed, new targeted therapies and immunotherapeutic strategies have significantly changed the landscape of metastatic melanoma treatment, transforming the metastatic melanoma in an oncological model and in a prototype for innovative therapies [3]. Nevertheless, metastatic melanoma is one of the most drug-resistant cancers [4] that often acquires the capacity to evade the cytotoxic action of therapeutic options inducing the breakdown of cell death control such as the inactivation of p53 induced cell death pathways [5] and the activation of PI3K/AKT (Phosphatidylinositol 3-Kinase/transforming retrovirus, AKT8, isolated from a spontaneous thymoma of a stock A strain K mouse) induced cell survival pathways [6]. Therefore, with the aim to identify new potential therapeutic drugs suitable to overcome the intrinsic survival features acquired by melanoma cells, we studied the role of hydroxytyrosol [2-(3,4-dihydroxyphenyl) ethanol or 3-hydroxytyrosol] on the inhibition of human melanoma cell growth and survival. Hydroxytyrosol, oleuropein, and tyrosol are the major phenolic components of olive oil and since oleuropein is the higher phenolic component of olive fruit during the first stage of development [7], hydroxytyrosol is the major phenolic component of olive oil [8]. In particular, hydroxytyrosol is a product of the oleuropein hydrolysis that occurs during the ripening process of olive fruit (*Olea europaea* L., Oleaceae), the processing and storage of olive oil and the digestion process after olive oil ingestion [7]. Interestingly, olives and olive oil are crucial components of the Mediterranean diet [9] and their regular intake has been related to reduced risk of several chronic diseases such as cardiovascular, metabolic, and neurodegenerative diseases, cognitive dysfunctions, and cancer [10,11]. Indeed, among the olive oil phenols, the Hydroxytyrosol has the strongest antioxidant activity [12,13] and its protective effect is evident at very low concentrations in vitro for its ability to scavenge different oxidant chemical species to prevent DNA breaks induced by reactive oxygen species (ROS), but also to stimulate the synthesis and the activity of antioxidant enzymes [14]. Notwithstanding its antioxidant activity, Hydroxytyrosol can also interfere with differentiation, growth, proliferation and invasiveness of several cancers such as colorectal, pancreatic, skin, breast, lung, blood, bladder, prostate, gastric, and brain cancers regulating the pathways involved in the control of these processes [15,16,17,18], but through molecular mechanisms unrelated to its antioxidant activity [19]. In particular, Hydroxytyrosol could alter the oxidative equilibrium acting either as antioxidant or as pro-oxidant showing at low concentrations its potent antioxidant activity [14] and in long-term treatments at high concentrations, the production of ROS and the consequent induction of cell death for apoptosis [19]. Indeed, the pro-oxidant activity of Hydroxytyrosol mediated by the generation of hydrogen peroxide is probably due to auto-oxidation process that is the most common anticancer mechanism of Hydroxytyrosol [20]. This unexpected behaviour of Hydroxytyrosol has been extensively studied and in particular has been reported its ability to inhibit proliferation and induce the death for apoptosis of several tumour cell lines such as leukaemia [20], colon [19,20], prostate [20], pancreatic [17], breast [20,21,22], hepatic [23], and thyroid cancer cell lines [24], suggesting its possible wide use in cancer prevention and treatment. In particular, hydroxytyrosol can induce apoptosis through the release of cytochrome c from mitochondria and the activation of caspases, but also the cell cycle arrest, lowering the cyclin-dependent kinases (CDKs), inhibiting ERK 1/2-cyclin D1 and PI3K/AKT pathways. Moreover, hydroxytyrosol can inhibit in vitro the functions of Fatty Acid Synthase (FAS), B-cell lymphoma 2 (Bcl-2), cAMP Response Element-Binding (CREB), p38, Extracellular-signal-Regulated Kinase 1/2 (ERK 1/2), c-Jun N-terminal Kinase (JNK) as well as the expression of Nuclear Factor kappa-light-chain-enhancer of activated B cells (NF-kB) and Epidermal Growth Factor Receptor (EGFR) [16,17]. The in vivo experiments and epidemiological data confirmed the in vitro results, providing support to the known properties of olive oil phenols to inhibit the formation as well as the progression of cancers [15,25]. The olive oil is also traditionally used for topical treatment of the skin such as dermatitis, eczema, and photo aging [26] and the alginate bilayer films containing hydroxytyrosol have been proposed as topical chemotherapy for the treatment of skin cancer [27]. Furthermore, in several human melanoma cell lines, the olive oil polyphenol oleocanthal induces the death for apoptosis through the cleavage of caspase-9, -3 and poly (ADP-ribose) polymerase (PARP), as well as inhibiting AKT and ERK 1/2 phosphorylation. Moreover, oleocanthal has been shown to inhibit xenograft-induced melanoma growth, proliferation, and angiogenesis and also to significantly reduce the metastatic dissemination of melanoma [28]. Therefore, in the light of all these data, in this paper we improved and increased this field of research showing that hydroxytyrosol treatment remarkably reduces the cell viability of melanoma cells inducing the death for apoptosis. In particular, the activation of caspase-9 and caspase-3, as well as the cleavage of PARP that we showed, demonstrate the hydroxytyrosol mediated activation of intrinsic apoptotic pathway in treated melanoma cells. Notably, in melanoma cells treated with hydroxytyrosol, we also reported a significant increase of p53 and γH2AX expression and a significant decrease of AKT expression. Finally, we reported intracellular reactive oxygen species (ROS) increase as well as the inhibition of cell colony formation and therefore of melanoma metastatic progression. Indeed, hydroxytyrosol is widely accepted in cancer treatment for its anti-tumour properties and low toxicity; therefore, the results obtained suggest that the hydroxytyrosol, for its role in the inhibition of melanoma growth, might provide the basis for an effective treatment of this highly invasive tumour as potential chemotherapeutic agent.

## 2. Results

### 2.1. The Hydroxytyrosol Treatment Induces the Death of Melanoma Cells

**Figure 1:**
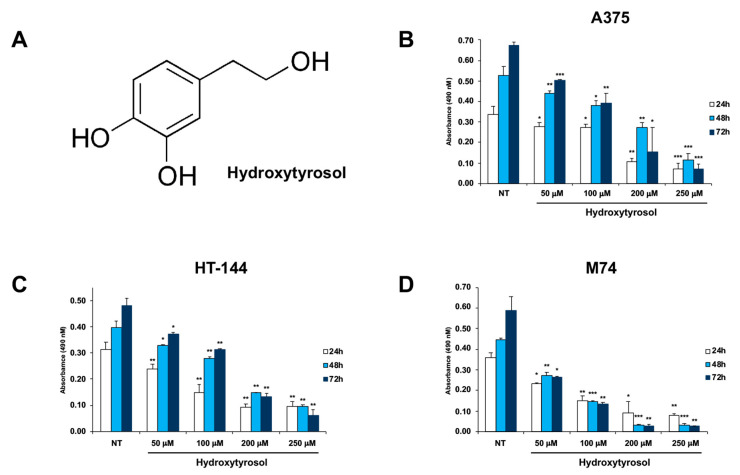
MTS assay of hydroxytyrosol treated melanoma cells. (**A**) Structure of hydroxytyrosol. A375 (**B**), HT-144 (**C**), and M74 (**D**) melanoma cells were treated with 50 μM, 100 μM, 200 μM, and 250 μM of hydroxytyrosol for 24 h, 48 h, and 72 h. The mean values of almost three different experiments performed in triplicate are reported in the histograms (**B**–**D**). The error bars indicate the standard deviation and statistical significance was analysed by Student’s *t*-test: * *p* < 0.05 was considered significant; ** *p* < 0.01 highly significant; *** *p* < 0.001 very highly significant.

The cytotoxicity of hydroxytyrosol was studied in A375, HT-144, and M74 human melanoma cells treated for 24 h, 48 h, and 72 h with hydroxytyrosol (Figure 1A) ranging from 50 μM to 250 μM, and the cellular growth of treated and untreated cells was analysed through 3-(4,5-dimethylthiazol-2-yl)-5-(3-carboxymethoxyphenyl)-2-(4-sulfophenyl)-2H–tetrazolium (MTS) assay (Figure 1B–D). The results of these experiments show that the hydroxytyrosol treatment induces a dose- and time-dependent decrease of cellular viability of A375, HT-144, and M74 melanoma cells (Figure 1B–D) compared to untreated cells (NT). In particular, the treatment of melanoma cells with 200 μM and 250 μM of hydroxytyrosol induces the inhibition (Figure 1B–D) of the cellular proliferation to a greater extent after 72 h of treatment, compared to untreated cells (NT).

### 2.2. The Hydroxytyrosol Induces the Death for Apoptosis of Human Melanoma Cells

**Figure 2:**
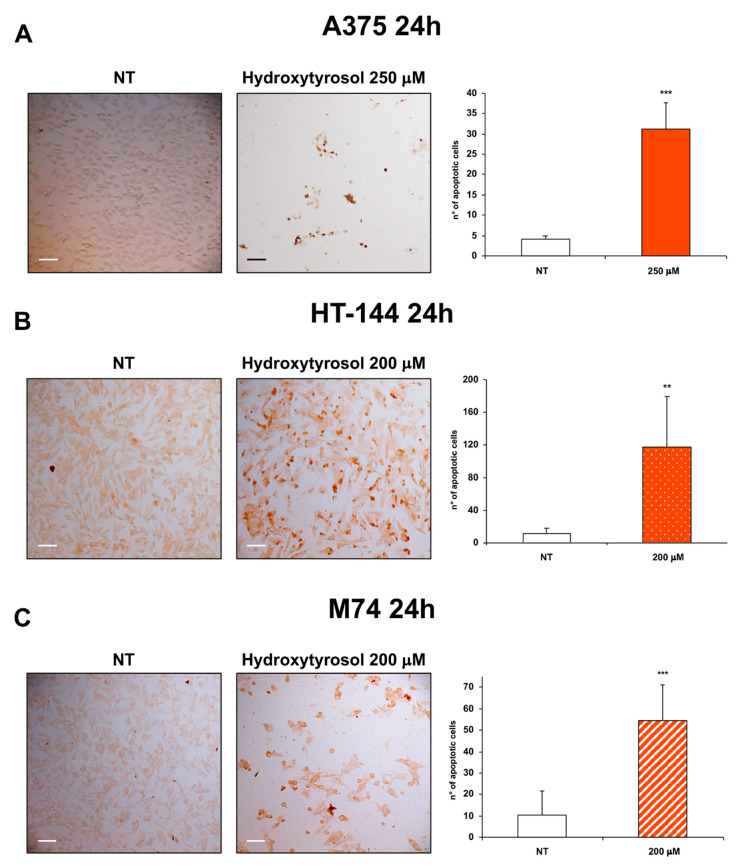
TUNEL analysis of hydroxytyrosol treated melanoma cells. A375 (**A**) cells were treated with 250 μM of hydroxytyrosol, HT-144 (**B**) and M74 (**C**) cells were treated with 200 μM of hydroxytyrosol for 24 h. Colorimetric TUNEL staining was used to analyse the apoptotic cells of A375 (**A**), HT-144 (**B**), and M74 (**C**) untreated (left panels) and treated (middle panels) cells. Images were acquired with a Zeiss Axioskop 2 Plus microscope using a ×10 objective. Scale bar: 100 μm. The number of stained apoptotic cells were reported in the histograms (right panels). The data are representative of almost three different experiments, the error bars indicate the standard deviation, and statistical significance was analysed by the Student’s *t*-test: ** *p* < 0.01 highly significant; *** *p* < 0.001 very highly significant.

To further investigate the effects of hydroxytyrosol treatment in melanoma cells, we analysed by terminal deoxynucleotidyl transferase-mediated dUTP nick-end labeling (TUNEL) assay the DNA fragmentation, a hallmark of apoptosis, of treated and untreated melanoma cells. In particular, the TUNEL staining of A375 cells treated with 250 μM of hydroxytyrosol and of HT-144 and M74 melanoma cells treated with 200 μM of hydroxytyrosol for 24 h, shows a markedly increased amount of DNA fragmentation compared to untreated cells (Figure 2A–C).

**Figure 3:**
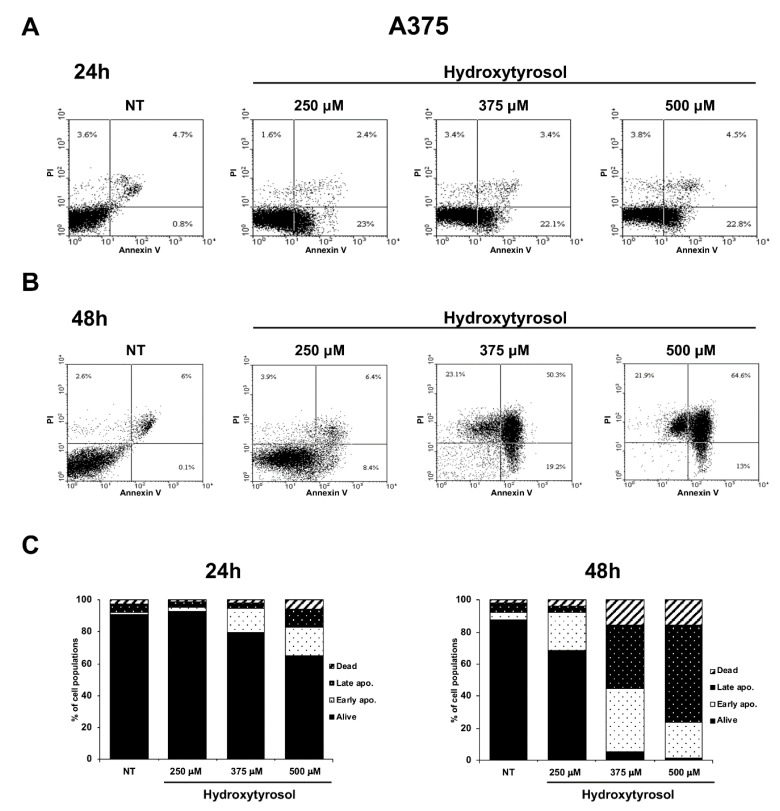
Flow cytometric analysis of A375 melanoma cells treated with hydroxytyrosol. A375 cells untreated (NT) or treated for 24 h (**A**) and 48 h (**B**) with 250 μM, 375 μM, and 500 μM of hydroxytyrosol were analysed by annexin V-FITC/PI staining through flow cytometry. The percentage of cells alive (lower left quadrant), early apoptotic cells (lower right quadrant), late apoptotic cells (upper right quadrant), and necrotic cells (upper left quadrant) were reported in flow cytometric graphics. The graphics of flow cytometric analysis are representative of several independent experiments. The percentage of early apoptotic, late apoptotic, and necrotic A375 cells, untreated (NT) or treated for 24 h (left graphic) and for 48 h (right graphic) with hydroxytyrosol, as mean values of several different experiments are reported in the histograms (**C**).

**Figure 4:**
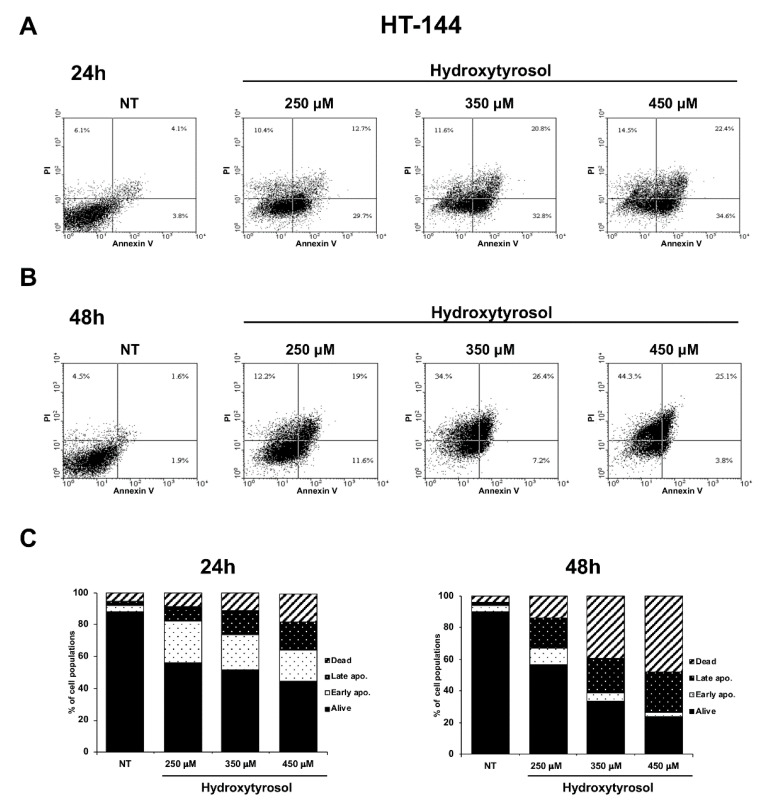
Flow cytometric analysis of HT-144 melanoma cells treated with hydroxytyrosol. HT-144 cells untreated (NT) or treated for 24 h (**A**) and 48 h (**B**) with 250 μM, 350 μM, and 450 μM of hydroxytyrosol were analysed by annexin V-FITC/PI staining, through flow cytometry. The percentage of cells alive (lower left quadrant), early apoptotic cells (lower right quadrant), late apoptotic cells (upper right quadrant), and necrotic cells (upper left quadrant) were reported in flow cytometric graphics. The data reported are representative of several independent experiments of flow cytometric analysis. The percentage of early apoptotic, late apoptotic, and necrotic HT-144 cells untreated (NT) or treated for 24 h (left graphic) and 48 h (right graphic) with hydroxytyrosol as mean values of several different experiments are reported in the histograms (**C**).

Apoptosis is a form of programmed cell death whose dysregulation underlies physiological but also pathological processes including tumorigenesis [29]. In particular, in the early apoptosis, the lipid phosphatidylserine (PS) is translocated from the inner to the outer layer of the membrane allowing annexin V staining of PS; furthermore, in the late apoptosis and during necrosis some pores appearing in the cell membranes allow Propidium Iodide (PI) binding of DNA [30]. Therefore, to demonstrate the hydroxytyrosol induced apoptotic death in treated melanoma cells, A375 cells were treated with 250 μM, 375 μM, and 500 μM and HT-144 cells with 250 μM, 350 μM, and 450 μM of hydroxytyrosol for 24 h and 48 h. The apoptosis/necrosis differential assessment of treated and untreated melanoma cells was analysed by annexin V-FITC/PI staining coupled with flow cytometry. The results of these experiments show that the treatment with hydroxytyrosol for 24 h and 48 h significantly increases the number of A375 and HT-144 apoptotic cells (Figure 3 and Figure 4). In particular, the populations of A375 cells in early and late apoptotic stage reach the maximum after 500 μM treatment with hydroxytyrosol for 24 h (17.55% and 11.25%, respectively) compared to untreated cells (1.0% and 5.4%) (Figure 3A,C, left panel) and for 48 h (22.50% and 60.70%, respectively) compared to untreated cells (4.30% and 6.20%) (Figure 3B,C, right panel). The rate of necrotic A375 cells ranges from 2.8% of untreated cells to 6.1% of cells treated for 24 h (Figure 3A,C, left panel) with 500 μM of hydroxytyrosol and from 1.85% of untreated cells to 15.45% and 15.55% of cells treated for 48 h with 375 μM and 500 μM of hydroxytyrosol respectively (Figure 3B,C, right panel).

Moreover, the populations of HT-144 cells in early and late apoptotic stage reach the maximum after 450 μM treatment with hydroxytyrosol for 24 h (19.25% and 17.85%, respectively) compared to untreated cells (3.55% and 3.20%) (Figure 4A,C, left panel) and for 48 h (2.95% and 26.55%, respectively) compared to untreated cells (3.35% and 2.15%) (Figure 4B,C, right panel). Finally, the rate of necrotic HT-144 cells ranges from 5.05% of untreated cells to 8.4%, 11.4%, and 17.35% of cells treated for 24 h with 250 μM, 350 μM, and 450 μM of hydroxytyrosol respectively (Figure 4A,C, left panel) and from 4.10% of untreated cells to 14.00%, 39.15%, and 47.90% of cells treated for 48 h respectively (Figure 4B,C, right panel). Therefore, these results show that hydroxytyrosol induces the death of melanoma cells, essentially activating the early apoptotic pathway with the exception of A375 cells treated for 48 h with 500 μM of hydroxytyrosol (60.70% of cells in late apoptosis) and of HT-144 cells treated for 48 h with 350 μM and with 450 μM of hydroxytyrosol (39.15% and 47.90% of cells in necrosis), (Figure 3 and Figure 4). The results of these experiments collectively show that hydroxytyrosol exert its anti-proliferative effect in melanoma cells essentially through the induction of apoptotic pathways (Figure 2, Figure 3 and Figure 4).

### 2.3. The Hydroxytyrosol Activates the Apoptotic Pathways in Melanoma Cells

**Figure 5:**
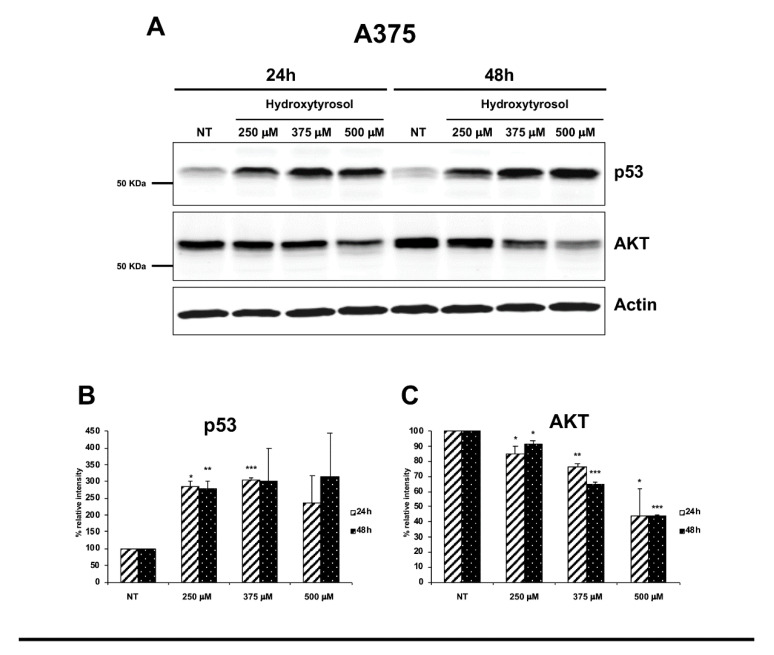

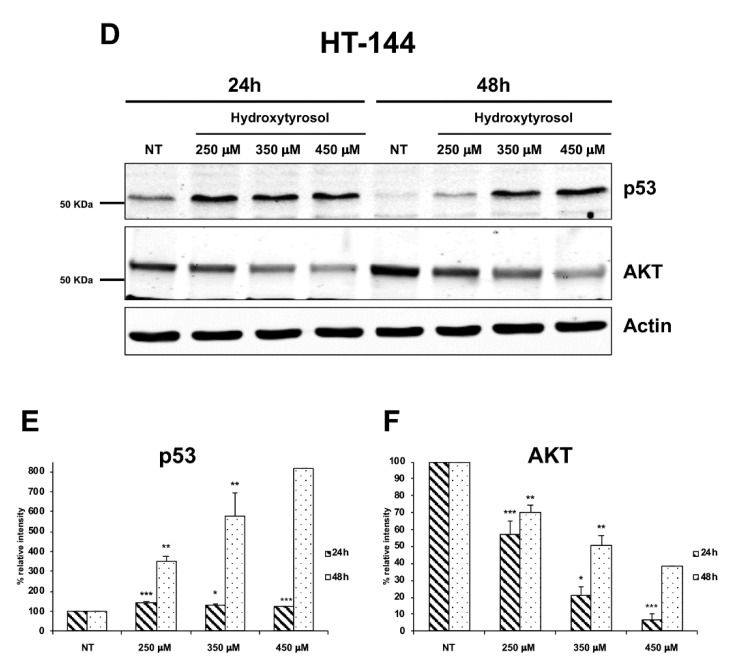
Expression of p53 and AKT proteins in hydroxytyrosol treated melanoma cells. A375 cells (**A**–**C**) were treated with 250 μM, 357 μM, and 500 μM of hydroxytyrosol and HT-144 cells (**D**–**F**) were treated with 250 μM, 350 μM, and 450 μM of hydroxytyrosol for 24 h and 48 h. The expression of p53, (**A**,**D**, upper panels, **B**,**E**) and AKT signalling proteins, (**A**,**D**, middle panels, **C**,**F**), was analysed in A375 and HT-144 cell lines in response to the treatment with hydroxytyrosol through western blot experiments. The equal protein loading was confirmed by the analysis of β-actin expression, (**A**,**D**, lower panels). The data are representative of several western blot experiments. The error bars indicate the standard deviation. Statistical significance was analysed by Student’s *t*-test: * *p* < 0.05 was considered significant; ** *p* < 0.01 highly significant; *** *p* < 0.001 very highly significant.

To further understand the mechanisms used by hydroxytyrosol to induce the death of treated melanoma cells, we analysed the expression of several pro-apoptotic and survival proteins such as p53 signalling protein, pro-caspases-9 and -3, PARP-1, the serine-139-phosphorylated form of histone variant H2AX (γH2AX), and the signalling protein AKT. In particular, we treated A375 cells with 250 μM, 375 μM, and 500 μM of hydroxytyrosol and HT-144 cells with 250 μM, 350 μM, and 450 μM of hydroxytyrosol for 24 h and 48 h and we analysed the expression of pro-apoptotic and survival proteins through western blot experiments. The results obtained show in both treated melanoma cell lines, the increase, time, and concentration dependent, of p53 expression (Figure 5A,D, upper panels; B and E) as well as the decrease, time, and concentration dependent of AKT signalling protein expression (Figure 5A,D, middle panels; C and F) compared to untreated cells (NT). Indeed, the tumour suppressor p53 and the oncoprotein AKT regulate the tumorigenesis in opposite way, promoting and suppressing the cellular growth respectively. Moreover, p53 and AKT signalling proteins can inhibit each other with the aim to balance survival and death signals [5].

**Figure 6:**
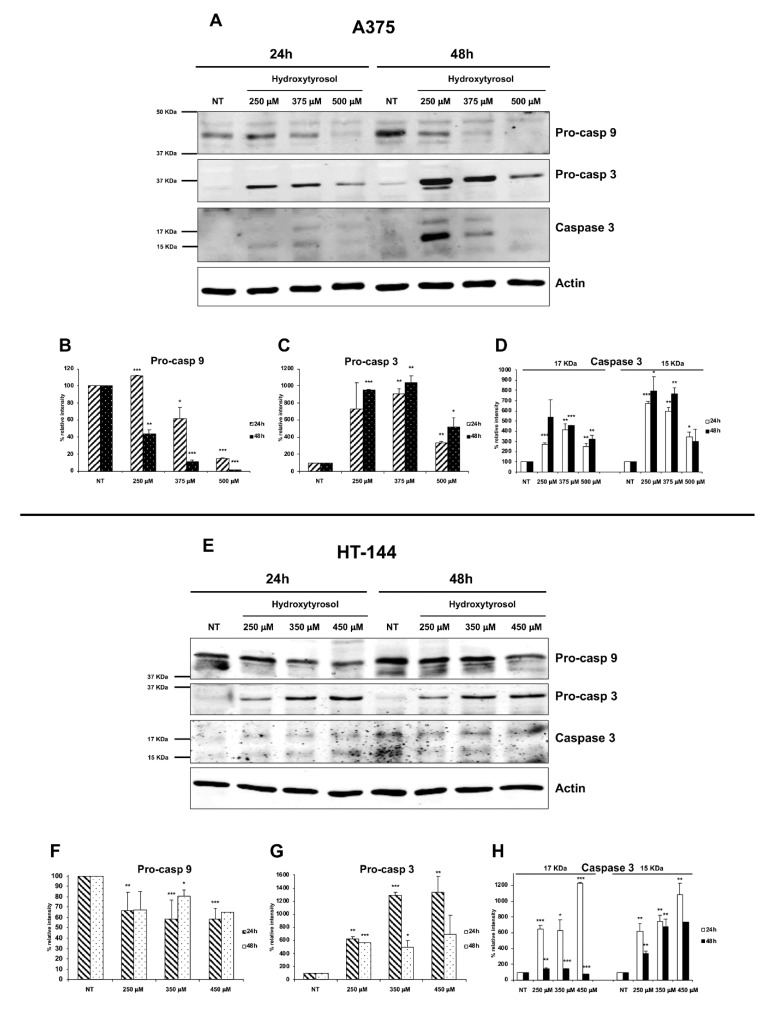
Expression of pro-caspase 9, pro-caspase 3, and caspase 3 in hydroxytyrosol treated melanoma cells. A375 cells (**A**–**D**) were treated with 250 μM, 375 μM, and 500 μM of hydroxytyrosol and HT-144 cells (**E**–**H**) were treated with 250 μM, 350 μM, and 450 μM of hydroxytyrosol for 24 h and 48 h. The expression of pro-caspase 9 (**A**,**E**, upper panels, **B**,**F**), pro-caspase 3 (**A**,**E**, second panels, **C**,**G**), and caspase 3 (**A**,**E**, third panels, **D**,**H**) was analysed in A375 and HT-144 cell lines in response to the treatment with hydroxytyrosol through western blot experiments. The equal protein loading was confirmed by the analysis of β-actin expression (**A**,**E**, lower panels). The data are representative of several western blot experiments. The error bars indicate the standard deviation. Statistical significance was analysed by Student’s *t*-test: * *p* < 0.05 was considered significant; ** *p* < 0.01 highly significant; *** *p* < 0.001 very highly significant.

Furthermore, the analysis of the expression, by western blot experiments, of initiator caspase-9 and of effector caspase-3, shows in both A375 and HT-144 treated cells, a concentration dependent decrease of caspase-9 precursor expression (Figure 6A,E, upper panels; B and F) and the increase of both precursor and activated forms of effector caspase-3, compared to untreated cells (NT) (Figure 6A,E, middle and lower panels; C, D, G, and H).

**Figure 7:**
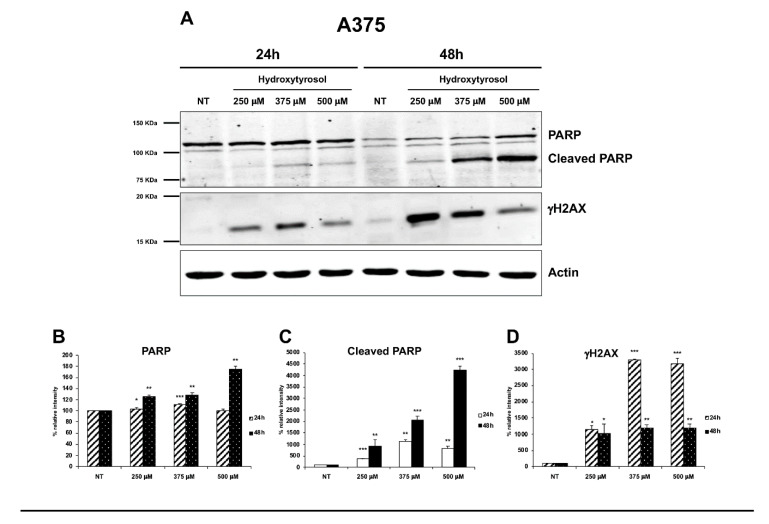

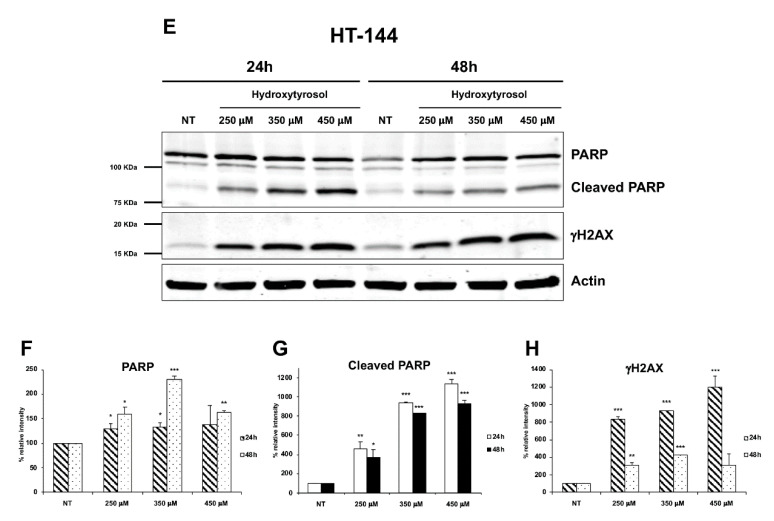
Expression of PARP-1 and γH2AX in hydroxytyrosol treated melanoma cells. A375 cells (**A**–**D**) were treated with 250 μM, 375 μM, and 500 μM of hydroxytyrosol and HT-144 cells (**E**–**H**) were treated with 250 μM, 350 μM, and 450 μM of hydroxytyrosol for 24 h and 48 h. The expression of PARP-1 (**A**,**E**, upper panels, **B**,**F**), of the cleaved form of PARP-1 (**A**,**E,** upper panels, **C**,**G**), and of γH2AX (**A**,**E**, middle panels, **D**,**H**), was analysed in A375 and in HT-144 cell lines in response to the treatment with hydroxytyrosol through western blot experiments. The equal protein loading was confirmed by the analysis of β-actin expression (**A**,**E**, lower panels). The data are representative of several western blot experiments. The error bars indicate the standard deviation. Statistical significance was analysed by Student’s *t*-test: * *p* < 0.05 was considered significant; ** *p* < 0.01 highly significant; *** *p* < 0.001 very highly significant.

Moreover, using an antibody direct against the full-length and the cleaved forms of PARP-1, we show that hydroxytyrosol treatment leads to a significant increase, time, and concentration dependent, of the cleaved form of PARP-1 in both A375 and HT-144 treated cells, compared to untreated cells (NT) (Figure 7A,E, upper panels; B, C, F, and G). PARP-1 is a nuclear enzyme that plays a pivotal role in the repair of DNA breaks [31], and its cleavage is an apoptotic marker and an indicator of caspase activation [32]. Finally, to further evaluate hydroxytyrosol induced DNA damage, we analysed in treated A375 and HT-144 cells the expression of a central player of DNA damage response (DDR) and in particular of the DNA double strand breaks (DSB) damage response, the phosphorylate form of histone H2AX (γH2AX) [33]. Interestingly, in treated A375 and HT-144 melanoma cells, we show the concentration dependent increase of γH2AX expression (Figure 7A,E, lower panels; D and H), compared to untreated cells (NT), strongly suggesting that hydroxytyrosol induces DNA double strand breaks in treated melanoma cells. In conclusion, the activation of pro-apoptotic proteins such as p53, caspases 9 and 3, and the down regulation of survival proteins such as PARP-1 and AKT as well as the increased expression of γH2AX protein, strongly suggest that hydroxytyrosol treatment induces the death for apoptosis through the activation of intrinsic apoptotic pathway in melanoma cells.

### 2.4. Effects of Hydroxytyrosol Treatment on the Production of Intracellular Reactive Oxygen Species (ROS) in Melanoma Cells

**Figure 8:**
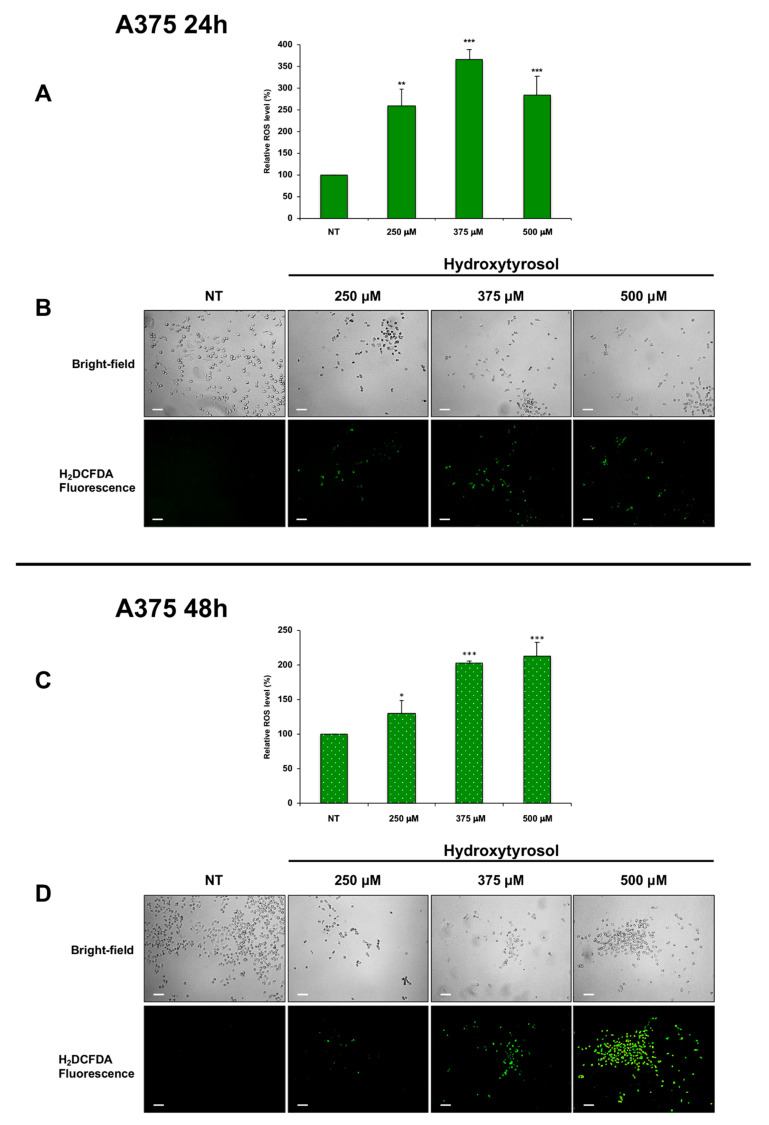
Amount of ROS in A375 cells treated with hydroxytyrosol. A375 melanoma cells were treated with 250 μM, 375 μM, and 500 μM of hydroxytyrosol for 24 h (**A**,**B**) and 48 h (**C**,**D**). The green-fluorescent emission of DCF was quantified (**A**,**C**) and was analysed (**B**,**D**) through an inverted fluorescence microscope. The intensity of fluorescence that is directly proportional to hydrogen peroxide concentration was reported in the histograms (**A**,**C**) as the mean values of ROS acquired from almost three different experiments performed in triplicate and is expressed as a percent of ROS produced in unstimulated cells. The error bars indicate the standard deviation, statistical significance was analysed by Student’s *t*-test: * *p* < 0.05 was considered significant; ** *p* < 0.01 highly significant; *** *p* < 0.001 very highly significant. The bright-field and fluorescence photomicrography of treated and untreated A375 cells stained with H_2_DCFDA probe are reported in (**B**,**D**). Images were acquired with an inverted Leica fluorescence microscope using a ×10 objective. Scale bar: 100 μm. The images are representative of almost three different experiments.

**Figure 9:**
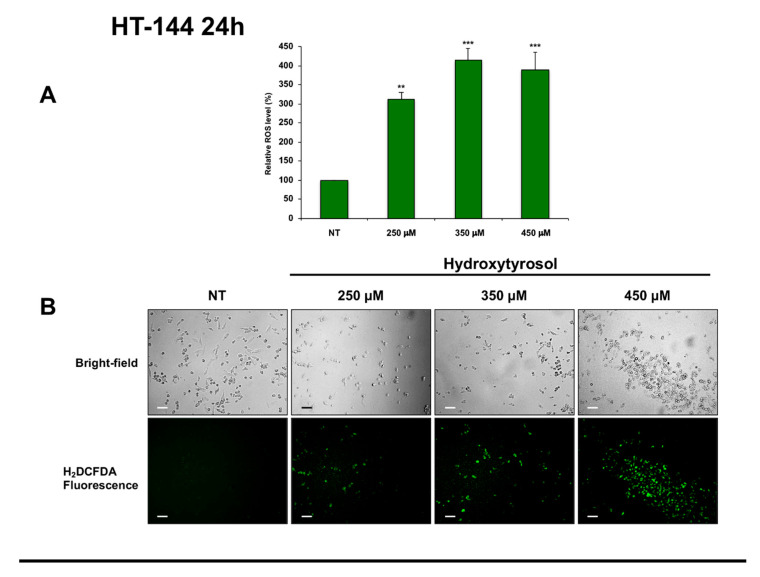

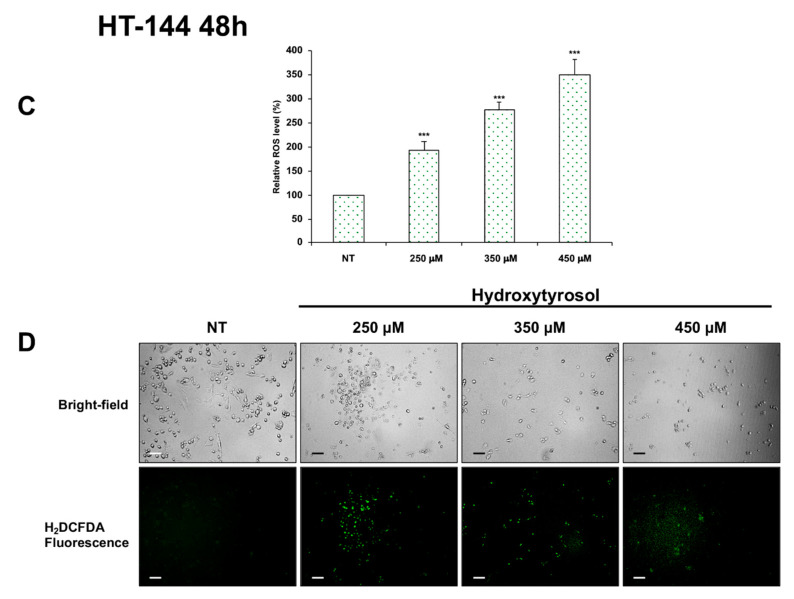
Amount of ROS in HT-144 cells treated with hydroxytyrosol. HT-144 melanoma cells were treated with 250 μM, 350 μM, and 450 μM of hydroxytyrosol for 24 h (**A**,**B**), and 48 h (**C**,**D**). The green-fluorescent emission of DCF was quantified (**A**,**C**) and was analysed (**B**,**D**) through an inverted fluorescence microscope. The intensity of fluorescence that is directly proportional to hydrogen peroxide concentration was reported in the histograms (**A**,**C**) as the mean values of ROS acquired from almost three different experiments performed in triplicate and is expressed as a percent of ROS produced in unstimulated cells. The error bars indicate the standard deviation, statistical significance was analysed by Student’s *t*-test: ** *p* < 0.01 highly significant; *** *p* < 0.001 very highly significant. The bright-field and fluorescence photomicrography of treated and untreated HT-144 cells stained with H_2_DCFDA probe are reported in (**B**,**D**). Images were acquired with an inverted Leica fluorescence microscope using a ×10 objective. Scale bar: 100 μm. The images are representative of almost three different experiments.

Interestingly, ROS production eliciting oxidative stress and consequently prominent DNA damage is an emerging approach for cancer therapy [34]. Therefore, to verify if hydroxytyrosol exerts its cytotoxic effects in melanoma cells via reactive oxygen species production, we analysed the amount of intracellular ROS in untreated cells and in A375 cells treated with 250 μM, 375 μM, and 500 μM of hydroxytyrosol (Figure 8) as well as in HT-144 cells treated with 250 μM, 350 μM, and 450 μM of hydroxytyrosol for 24 h and 48 h (Figure 9). The results of these experiments show that the amount of intracellular ROS after 24 h of treatment reaches the maximum in A375 cells treated with 375 μM of hydroxytyrosol (366.27%) (Figure 8A,B) and in HT-144 cells treated with 350 μM of hydroxytyrosol (415.37%) (Figure 9A,B), compared to untreated cells. Furthermore, the analysis of intracellular ROS after 48 h of treatment shows that the level of ROS reaches the maximum in A375 cells treated with 500 μM of hydroxytyrosol (212.75%) (Figure 8C,D) and in HT-144 cells treated with 450 μM of hydroxytyrosol (350.77%) (Figure 9C,D), compared to untreated cells. Therefore, the increase of intracellular ROS in A375 and HT-144 melanoma cells treated with hydroxytyrosol suggests that the cytotoxic functions of hydroxytyrosol are modulated by ROS production that could be involved in hydroxytyrosol induced DNA damage and apoptosis.

### 2.5. Cell Colony Formation Assay of Treated Melanoma Cells

**Figure 10:**
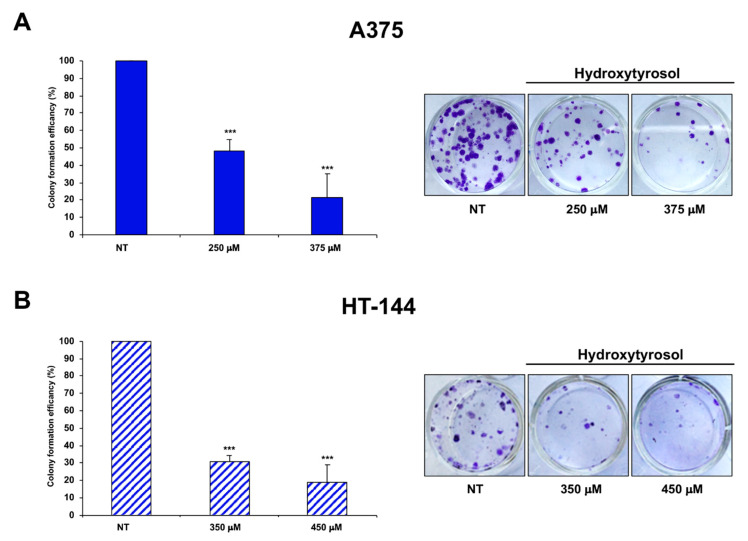
Cell colony assay of hydroxytyrosol treated melanoma cells. A375 cells (**A**) were treated with 250 μM and 375 μM of hydroxytyrosol and HT-144 cells (**B**) were treated with 350 μM and 450 μM of hydroxytyrosol for 24 h. The colony formation efficiency of treated cells is the colony numbers formed by treated cells expressed as a percent of the colonies formed by untreated cells (NT) and is reported in the histograms (**A**,**B**, left panels), as mean values of almost three different experiments. The error bars indicate the standard deviation and statistical significance was analysed by Student’s *t*-test: *** *p* < 0.001 very highly significant. In (**A**,**B**), right panels, bright-field photography of treated and untreated A375 (**A**) and HT-144 (**B**) cell colony are reported. The images are representative of almost three different experiments.

Furthermore, we analysed the ability to form colonies of A375 cells treated for 24 h with 250 μM and 375 μM of hydroxytyrosol and of HT-144 cells treated for 24 h with 350 μM and 450 μM of hydroxytyrosol (Figure 10A,B). Interestingly, hydroxytyrosol treatment inhibits the ability of melanoma cells to form cell colonies, in particular A375 melanoma cells treated with 250 μM and with 375 μM of hydroxytyrosol displayed 48.48% and 21.53% of cell colony number respectively, compared to untreated cells (Figure 10A). HT-144 cells treated with 350 μM and with 450 μM of hydroxytyrosol displayed 30.83% and 18.57% of cell colony number respectively, compared to untreated cells (Figure 10B). Therefore, our results show that the treatment of melanoma cells with hydroxytyrosol reduces the cell viability and the survival rate of melanoma cells. 

## 3. Discussion

The growing understanding of the molecular pathways involved in the metastatic transformation of melanoma and the knowledge acquired on the ability of immune systems to fight cancer have led to the amazing development of targeted therapies and immunotherapeutic strategies [3]. Indeed, these new therapeutic options changed the landscape of metastatic melanoma treatments, but unfortunately they also induce cancer drug-resistance and significant side effects in melanoma patients [3,35]. However, the available chemotherapeutic drugs such as alkylating agents, dacarbazine, and temozolomide play a very limited effect on the treatment of metastatic melanoma [36] and are associated with a reduced overall response and generally low survival rates of melanoma patients [37]. Notwithstanding, a heterogeneous class of natural compounds with different chemical structures such as alkaloids, hydroxycinnamic acids, polyphenols, terpenoids, and tocotrienols exerts significant beneficial effects targeting several pathways involved in tumorigenesis and in tumour progression of melanoma [38]. In addition, the phenol components of olive oil, oleuropein, hydroxytyrosol, and tyrosol have been proposed to provide protection against chronic diseases such as cancer [10,11]. Therefore, with the aim to identify new therapeutic approaches for melanoma, we studied the role of the major phenolic component of olive oil, the hydroxytyrosol, on the inhibition of human melanoma cell growth. Indeed, the lack of hydroxytyrosol toxicity in non-malignant cells such as keratinocytes [39,40], fibroblasts [39], liver cells [23], colon epithelial cells [19], and PBMC (Peripheral Blood Mononuclear Cells) [41] also used at high concentrations as well as its ability to modulate several signalling pathways and to inhibit differentiation, growth, proliferation, and invasiveness of several cancers [15,16,17,18] makes hydroxytyrosol the ideal candidate for the treatment of this highly metastatic cancer. Therefore, in hydroxytyrosol treated melanoma cells we report a dose-dependent induction of the intrinsic apoptotic pathway through the activation of pro-apoptotic proteins such as p53 and caspases-9 and -3, the down regulation of survival proteins such as PARP and AKT, the increased expression of γH2AX protein, as well as the inhibition of cell colony formation. Indeed, in stress conditions correlated with DNA damage or chromosomal aberrations, the expression and activation of p53 increase markedly, improving its ability to modulate gene expression and cellular viability. In particular, in response to potent stress signals and irreparably damaged DNA, p53 activates the apoptotic pathways, thereby preventing the expansion of damaged cells into a large population of tumorigenic progeny [5]. Otherwise, AKT is a pro-survival and anti-apoptotic kinase that controls several downstream substrates and cellular pathways involved in the regulation of metabolism, cell growth, proliferation, survival, apoptosis, and angiogenesis, which overlap and cross-talk with the functions of additional cellular signalling networks [42]. Interestingly, tumour suppressor p53 and oncoprotein AKT regulate the cellular growth in opposite ways and the mutual regulatory crosstalk between these key signalling proteins regulates the cellular life and death [5]. Therefore, in hydroxytyrosol treated melanoma cells, the increased expression of tumour suppressor p53 and the decreased expression of oncoprotein AKT could be induced by an irreparable DNA damage that ultimately triggers the death of treated melanoma cells by the activation of apoptotic pathways. DNA damages, induced by reactive oxygen species, ionizing radiation, or genotoxic compounds trigger a cascade of DNA repair processes at the site of DNA damage [43] such as the phosphorylation of histone H2AX (γH2AX), a marker of DNA double strand breaks damage [33], or the activation of PARP-1 following its binding to DNA damaged [44]. Indeed, PARP-1 is involved in many different processes such as single and double strand breaks DNA damage repair, chromatin modification, transcriptional regulation, and cell death [44]. Nevertheless, the cleavage of PARP-1 by caspases, mainly upon irreparable DNA damage, is a hallmark of apoptosis and has a critical role in the regulation of cell death [45]. Moreover, the phosphorylation of H2AX is an early step of DNA damage response process, an elaborate signal transduction pathway activated in response to DNA damage, that induces a cellular response to allow DNA repair [46]. Therefore, in the light of the data reported, the hypothesis that hydroxytyrosol treatment of melanoma cells induces DNA double strand breaks damage, elicits not only the cleavage of PARP-1 by caspases, but also the increased expression of the phosphorylate form of H2AX. Indeed, the cellular consequences of hydroxytyrosol treatment are complex and concentration dependent, at lower concentrations hydroxytyrosol is an anti-oxidant compound for its ROS scavenger functions [14], but at higher concentrations hydroxytyrosol induces oxidative stress producing ROS and thus inducing the death for apoptosis of treated tumour cells [19]. Interestingly, ROS molecules also play a role, rather complex and paradoxical, in the cells. In particular, basal levels of ROS at physiologic conditions are essential to promote cell survival and proliferation [47], but in cancer cells, a moderate increase of ROS levels induces several biochemical and molecular alterations contributing to carcinogenesis, cancer cell survival, cancer progression, and resistance to chemotherapy [48]. Nevertheless, in cancer cells, the high increase of ROS levels induces DNA, proteins, and lipids damage as well as oxidative stress conditions and triggers the death of cancer cells through the activation of cell death pathways or the inhibition of cancer cell resistance to chemotherapy [48]. Therefore, in hydroxytyrosol treated melanoma cells, we showed a concentration and time dependent increase of intracellular ROS levels; thereby, our hypothesis is that hydroxytyrosol may induce the death for apoptosis of treated melanoma cells through the production of high amounts of ROS. Indeed, the oxidative stress conditions induced by the increase of ROS and the consequent DNA damage can elicit not only the activation of pro-apoptotic proteins such as p53 and caspases but also the increased expression of the phosphorylate form of H2AX and the caspase dependent cleavage of PARP-1. Finally, the inhibition of cell colony formation in treated melanoma cells strongly suggests that hydroxytyrosol induces irreversible cellular alterations, thus inhibiting the growth and the metastatic dissemination of melanoma. The reported results suggest that the strategy used by hydroxytyrosol to exert its cytotoxic function in melanoma involves the increase of intracellular ROS and therefore the ROS dependent regulation of the pathways involved in the activation of apoptosis and in the inhibition of melanoma growth. Therefore, the hydroxytyrosol treatment could deeply interfere with the melanoma progression as a promising therapeutic option for the treatment of melanoma.

## 4. Material and Methods

### 4.1. Cell Lines, Antibodies, and Reagents

A375 (RRID CVCL_0132; ATCC-CRL-1619), HT-144 (RRID CVCL_0318; ATCC-HTB-63), and M74 (kindly provided by Prof. C. Alcaide-Loridan, Institut Jacques Monod, Paris Diderot University, Paris, France) human melanoma cell lines were grown in RPMI 1640 supplemented with 10% FCS and 1% penicillin-streptomycin (10,000 U/mL and 10,000 μg/mL, respectively) in 5% CO_2_ at 37 °C. The hydroxytyrosol (H4291), purchased from Sigma-Aldrich (St Louis, MO, USA), was dissolved in Phosphate buffered saline solution (PBS) to obtain a 100 mM stock solution and further diluted in culture medium before each treatment. Mouse monoclonal antibody against p53 (DO-1, sc-126) and rabbit polyclonal antibodies against p-Histone H2A.X (γH2AX) (sc-101696) and caspase-9 (H-170, sc-8355) were purchased from Santa Cruz (Santa Cruz, CA, USA). Rabbit polyclonal antibodies direct against PARP-1 (BK9542S, CST), AKT (BK9272S, CST), and caspase-3 (BK9661S, CST) were obtained from Cell Signaling Technology (Leiden, The Netherlands). Anti-β-actin (AC-15, A5441) mouse monoclonal antibody was obtained from Sigma (St Louis, MO, USA). Infrared dye-conjugated IRDye800 anti-mouse (FE30926210) and anti-rabbit (FE30926211) (LI-COR Biosciences, Lincoln, NE, USA) as well as anti-mouse (A21058) and anti-rabbit (A21109) Alexa Fluor 680 conjugated (Molecular Probes, Eugene, OR, USA) were purchased as secondary antibodies. All other chemicals were of analytical grade and were purchased from Sigma Chemical Co. (St Louis, MO, USA), Merck (Darmstadt, Germany), or Thermo Fisher (Waltham, MA, USA).

### 4.2. Cell Viability Assay (MTS Assay)

A375 (0.5 × 10^4^ per well), HT-144 (1 × 10^4^ per well), and M74 (0.8 × 10^4^ per well) human melanoma cell lines were seeded in 96-well plates for 24 h. Therefore, the cells were treated with 50 μM, 100 μM, 200 μM, and 250 μM of hydroxytyrosol for 24 h, 48 h, and 72 h in triplicate, and the cell viability was determined using 3-(4,5-dimethylthiazol-2-yl)-5-(3-carboxymethoxyphenyl)-2-(4-sulfophenyl)-2*H*–tetrazolium (MTS) proliferation assay following the instructions of CellTiter-96 AQueous One Solution Cell Proliferation Assay (Promega, Madison, WI, USA). The plates were scanned at 490 nm in a 96-well plate reader after 30 min, 1 h, 1 h 30 min, and 2 h. Cell viability for each treatment was normalized against PBS treated cells.

### 4.3. DeadEnd™ Colorimetric TUNEL Assay

A375 (2 × 10^4^ per well), HT-144 and M74 (4 × 10^4^ per well) human melanoma cell lines were seeded in 8-well Lab-Tek II chamber slide for 24 h and then A375 cells were treated with 250 μM of hydroxytyrosol and HT-144 and M74 melanoma cells were treated with 200 μM of hydroxytyrosol for 24 h. After treatment, the medium was removed, the cells were washed with PBS, fixed with 4% paraformaldehyde in PBS at room temperature for 25 min, washed twice with PBS, and subjected to colorimetric TUNEL staining according to TUNEL protocols (Promega, Madison, WI, USA). The apoptotic cells were visualized using a under phase contrast microscope (Axioscope 2 plus, Zeiss, Oberkochen, Germany) using a ×10 objective and recorded by a digital camera system.

### 4.4. Flow Cytometry Assay

Semiconfluent A374 cells were treated with 250 μM, 375 μM, and 500 μM of hydroxytyrosol and HT-144 cells with 250 μM, 350 μM, and 450 μM of hydroxytyrosol for 24 h and 48 h. Therefore, floating and adherent cells were collected, washed twice with ice-cold PBS, and labelled with Annexin-V-Fluorescein and Propidium iodide (PI) using an Annexin-VFLUOS staining kit according to the instructions (Roche Diagnostic, Burgess Hill, West Sussex, UK). Therefore, the cells were acquired on a FACSCalibur™ flow cytometer, supported by CellQuest acquisition and data analysis software (Becton Dickinson, Mountain View, CA, USA).

### 4.5. Total Cell Extracts and Western Blot Analysis

Semiconfluent A374 cells were treated with 250 μM, 375 μM, and 500 μM of hydroxytyrosol and HT-144 cells with 250 μM, 350 μM, and 450 μM of hydroxytyrosol for 24 h and 48 h; therefore, the cells were lysed in RIPA buffer (50 mM Tris-HCl pH 8, 150 mM NaCl, 1% NP40, 0.5% DOC, 0.1% SDS) containing proteases and phosphatase inhibitors (4 mM PMSF and protease inhibitors cocktail, phosphatase inhibitors cocktail 2 and 3, Sigma). The protein concentration of cellular lysates was determined by Bradford protein assay (Bio-Rad laboratories GmbH, München, Germany). Furthermore, 20 μg or 40 μg of total cell extracts were resolved on sodium dodecyl sulphate-polyacrylamide gel electrophoresis (SDS-PAGE) and absorbed to nitrocellulose membrane (Hybond ECL, GE Healthcare, Biosciences, Pittsburgh, PA, USA) to perform western blot experiments as previously reported [49]. The membranes were processed and the protein bands were scanned and quantified by densitometric analysis, using Odyssey infrared imaging system (LI-COR Biosciences, Lincoln, NE, USA). The results of quantitative analysis are reported in the histograms as mean values of several western blot experiments and are expressed as a percent of unstimulated cells, normalized for actin amount.

### 4.6. Analysis of Intracellular Reactive Oxygen Species (ROS)

A375 cells (0.5 × 10^4^ per well) and HT-144 (0.8 × 10^4^ per well) cells, were grown in 96-well plates for 24 h, then the A375 cells were treated with 250 μM, 375 μM, and 500 μM and the HT-144 cells with 250 μM, 350 μM, 450 μM of hydroxytyrosol in triplicate for 24 h and 48 h. After treatment, the medium was removed, the treated and untreated cells were washed with PBS and labelled with 5 µM of carboxy-2′,7′-dichlorodihydro-fluorescein diacetate (carboxy-H_2_DCFDA) (Invitrogen™, Thermo-Fisher scientific, Waltham, MA, USA) for 5–10 min in the dark. The green-fluorescence emission (529 nm) of the oxidation product 2′,7′-dichlorofluorescein (DCF) after ROS production, was evaluated by fluorimeter (Microplate reader Wallac Victor 2 1420 Multilabel Counter, Perkin Elmer, Waltham, MA, USA) with a 488-nm excitation laser for quantitative investigations. The intensity of fluorescence is directly proportional to hydrogen peroxide concentration. Furthermore, an inverted fluorescence microscope (Leica, Wetzlar, Germany) with a ×10 objective and a digital camera system were used to document fluorescence images for qualitative investigations.

### 4.7. Cell Colony Assay

Semiconfluent A375 cells were treated with 250 μM and 375 μM and HT-144 cells with 350 μM and 450 μM of hydroxytyrosol for 24 h. Afterwards, A375 (0.18 × 10^4^ per well) and HT-144 (1.8 × 10^4^ per well) viable cells were seeded in a twelve well plate in triplicate. The treated and untreated cells were maintained in fresh medium for 7–14 days, were fixed in 100% methanol, and stained with 0.5% crystal violet in 20% methanol. Therefore, the plates were air dried, and the colonies were photographed using a digital camera and counted.

### 4.8. Statistical Analyses

The error bars indicate the standard deviation, statistical analyses were performed using Student’s *t*-test: * *p* < 0.05 was considered significant; ** *p* < 0.01 highly significant; *** *p* < 0.001 very highly significant.
